# Recombinant RGD-Apoptins Decrease Human Melanoma Cell Viability

**DOI:** 10.3390/ijms262412016

**Published:** 2025-12-13

**Authors:** Dmitriy Shirokov, Daria Lepekhina, Valentin Manuvera, Margarita Bogomiakova, Aleksandra Strokach, Anastasia Kazakova, Georgij Arapidi, Vassili Lazarev

**Affiliations:** 1 Lopukhin Federal Research and Clinical Center of Physical-Chemical Medicine of Federal Medical Biological Agency, Moscow 119435, Russiamargbog_5@mail.ru (M.B.); arapidi@gmail.com (G.A.); lazar0@mail.ru (V.L.); 2 K.I. Skryabin Moscow State Academy of Veterinary Medicine and Biotechnology, Moscow 109472, Russia; 3 Moscow Center for Advanced Studies, Moscow 123592, Russia; 4 Shemyakin-Ovchinnikov Institute of Bioorganic Chemistry of the Russian Academy of Sciences, Moscow 117997, Russia

**Keywords:** apoptin, RGD peptides, melanoma, αVβ3 integrins, therapeutic recombinant proteins, dual-specificity oncolytic proteins, MeWo, apoptosis, transcriptomics

## Abstract

Cutaneous melanoma is an extremely dangerous tumor disease with poor prognosis at advanced stages. Accounting for a small percentage of all skin tumors, malignant melanoma leads the mortality rate in this group of cancers. Clearly, the search for new drugs and therapeutic approaches for the treatment of cutaneous melanoma is a highly pressing issue in modern medicine. In this study, novel recombinant proteins with anti-melanoma activity, called RGD-apoptins, were produced in an *E. coli* expression system, and their properties were evaluated in human cell models. These chimeric proteins consist of two parts, each tumor-specific. One part of the chimeric molecule is the RGD peptide, which binds to αVβ3 integrins widely expressed on the surface of malignant melanocytes. The other part is the viral protein apoptin, known to induce programmed cell death in tumor cells but not in normal cells. This molecular design aims to enhance the specificity of potential therapeutic agent toward malignant melanoma cells while reducing cytolytic effects on healthy tissue. In a resazurin assay, RGD-apoptins decreased the viability of MeWo human melanoma cells and did not affect the viability of HaCaT human keratinocyte cell line and primary skin fibroblasts. Using an annexin V assay, we confirmed that malignant melanocytes death occurs via apoptosis. Transcriptomic analysis allowed us to dynamically evaluate the spectrum of differentially expressed genes 24 and 48 h after treating melanoma cells with recombinant RGD-apoptin.

## 1. Introduction

Melanoma is a tumor that occurs through the malignant transformation of pigment-producing cells, called melanocytes. These cells are primarily located in the basal layer of the epidermis, but are also occasionally found in the eyes and mucous membranes, resulting in the following incidence rates of melanocyte-derived tumors: 95% for cutaneous melanoma, 3.6% for uveal melanoma, and 1.4% for mucosal melanoma, respectively [1]. Due to the high metastatic potential of transformed melanocytes, late stages of melanoma are characterized by a poor prognosis. Although malignant melanomas account for only about 1% of all skin tumors, they are responsible for over 80% of deaths from this type of cancer [2]. More than 300,000 new cases of melanoma are diagnosed worldwide each year, and approximately 60,000 people die from this disease [1]. Clearly, the search for new drugs and therapeutic approaches for the treatment of malignant melanoma is a highly pressing issue in modern medicine.

αVβ3 integrins, which are heterodimeric cell adhesion molecules, are widely expressed on the plasma membrane of malignant melanocytes, prone to metastasis. However, the expression level of this integrin in normal melanocytes is quite low, allowing αVβ3 integrin to be considered a tumor-specific marker [3]. Integrins are notable for recognizing a very specific amino acid motif in their natural ligands (extracellular matrix proteins)—the amino acid sequence arginine–glycine–aspartic acid (RGD in single-letter code) [4]. The highly specific interaction of integrins with the RGD motif has led to the development of a wide range of therapeutic and diagnostic biomolecules that utilize this affinity binding [5,6]. Many of them are in various stages of clinical trials, and some drugs have been approved for therapeutic use [7]. Importantly, in addition to malignant melanocytes, αVβ3 integrins are expressed on the membranes of activated vascular endothelium cells, which makes it possible to kill two birds with one stone: to target RGD-based drugs not only to tumor cells but also to the vessels supplying them [8,9]. RGD-containing molecules can be used both as integrin blockers and as ligands for subsequent internalization into the cell. In the latter case, clustering of integrin receptors is usually required, which can be achieved, for example, by multimerization of RGD-containing ligands [10]. Previously, we demonstrated the successful delivery of recombinant tetrameric streptavidin fused with RGD peptides into human melanoma cells [11,12]. The integrin-targeting amino acid sequences we used were selected from peptide libraries using phage display by Holig et al. and contained an RGD motif flanked by two cysteines that stabilize the tripeptide by forming a disulfide bond [13].

In this study, we aimed to obtain and evaluate the properties of chimeric recombinant proteins consisting of two parts: an integrin-targeting (RGD peptide) and an oncolytic (apoptin protein). Apoptin is a small proline-rich protein of the chicken anemia virus (CAV) that can induce programmed cell death in transformed and malignant human cells, while remaining inactive in normal cells [14]. Structurally, apoptin consists of two domains: the N-terminal (amino acids 1 to 73) and the C-terminal (amino acids 74–121). The N-terminal domain contains a multimerization center (22–69) and a nuclear retention signal (33–46). The C-terminal domain contains a bipartite nuclear localization signal (82–88; 111–121), a nuclear export signal (97–105), and a key amino acid residue, threonine-108, the phosphorylation of which by tumor-specific kinase leads to inhibition of the nuclear export of apoptin and, consequently, to its accumulation inside the nucleus [15]. In normal cells, apoptin is not phosphorylated and therefore translocates back to the cytoplasm. Activation of programmed cell death by phosphorylated apoptin can occur through several mechanisms [16]. One of these mechanisms is the intrinsic Bcl-2-dependent apoptotic pathway with subsequent release of cytochrome c and AIF from mitochondria [17]. Another mechanism is mediated by the binding of apoptin multimers to DNA, which interferes with transcription, replication, and genome repair, ultimately leading to cell death via p53-independent apoptosis [18]. Some additional pathways of cell death induction by apoptin are discussed in scientific papers [15,16].

Chimeric (also known as fused) recombinant proteins have long been considered a promising class of drugs for use in anticancer therapy. The combination of two different proteins (or their parts) within a single molecule can enhance the effect of the recombinant drug, improve its pharmacokinetic properties, or target it to a tumor-specific receptor. A significant number of studies are devoted to the development and investigation of the potential therapeutic activity of fused proteins based on effector molecules of the human immune system. These can include combinations of variable and constant fragments of antibodies, T-cell receptors, and various cytokines. A review of research in this area is presented in the excellent paper by Weidle et al. [19]. Another example of chimeric molecules in oncotherapy are recombinant proteins consisting of bacterial toxins (Pseudomonas exotoxin (PE) and diphtheria toxin (DT)) fused to ligands for cell surface receptors (TGF-α, IL-2, IL-6) [20,21]. Finally, it is necessary to mention the use of proteins fused with short peptides that enable drug targeting and/or penetration into the cell. The RGD peptides mentioned above are just one of many examples of such molecules. Some of these peptides have also been used for the delivery of apoptin into cells. For instance, Guelen et al. created a chimeric protein consisting of apoptin and the protein transduction domain (PTD) of the HIV-1 TAT protein, demonstrating its efficient penetration into both tumor and normal cells [22]. Wang et al., in turn, used the hPP10 peptide from the human histone demethylase KDM4A for intracellular delivery of apoptin [23]. Furthermore, Hazrati et al. demonstrated the successful delivery of apoptin fused with the p28 peptide (from the bacterial protein azurin) into breast cancer cells [24].

**Figure 1:**
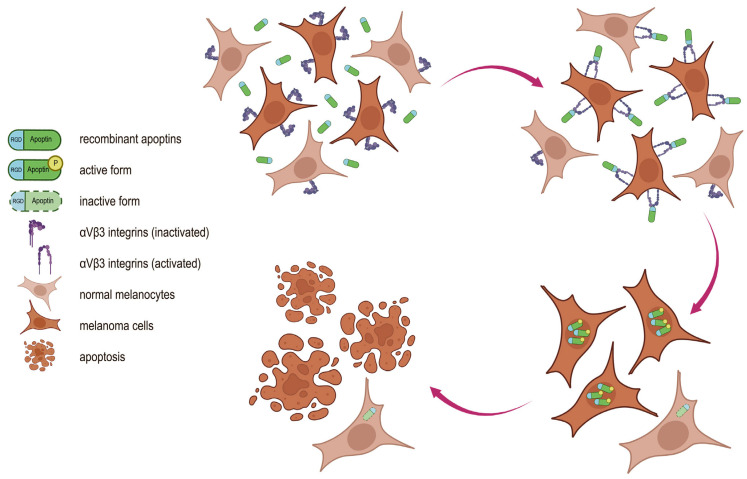
Proposed mechanism of action of recombinant RGD-apoptins. The dual specificity of the chimeric protein for tumor cells is conferred by its individual functional modules. The RGD peptide mediates binding of the chimeric protein to αVβ3 integrins, whose membrane expression is higher in malignant melanocytes than in normal melanocytes. Following internalization, apoptin selectively induces apoptosis in tumor cells while remaining inactive in normal cells.

It is widely known that the low selectivity of many anticancer drugs is associated with significant toxicity to normal tissues and often limits their use. Our proposed tumor-specific drug design has the potential to increase the selectivity of therapy for malignant cells. In this case, the choice between normal and tumor cells is a two-stage process (Figure 1). In the first stage (extracellular), preferential binding of the drug to the cancer cell is ensured by recognition of a tumor-specific receptor. In the second stage (intracellular), cancer cell death occurs through the action of a specific oncolytic molecule.

## 2. Results

### 2.1. Production of Recombinant Apoptins in E. coli

**Figure 2:**
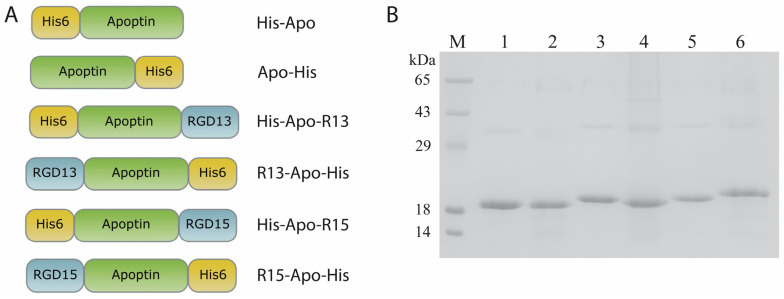
(**A**) Schemes of the recombinant RGD-apoptins generated in this study, His6—hexahistidine tag, apoptin—oncolytic protein, RGD13/RGD15—peptides containing the Arg-Gly-Asp (RGD) motif; protein names are indicated to the right of each schematic. (**B**) 15% SDS-PAGE analysis of purified and renatured RGD-apoptins. lane M—molecular weight marker (14 kDa, 18 kDa, 29 kDa, 43 kDa, 65 kDa), lane 1—His-Apo, lane 2—Apo-His, lane 3—His-Apo-R13, lane 4—R13-Apo-His, lane 5—His-Apo-R15, lane 6—R15-Apo-His.

The design of recombinant proteins based on apoptin involved the use of two RGD peptides: RGD13 (LSGCRGDCFQQ) and RGD15 (DGFPGCRGDCSQE), which have proven effective in binding to αVβ3 integrins [11,13]. Additional amino acid fragments and their location can significantly affect, firstly, the translation efficiency of the recombinant protein (and consequently, its production in heterologous expression systems), and secondly, the biological activity of the resulting polypeptide. For this reason, each RGD-apoptin was produced in two variants: with an N-terminal and a C-terminal location of the targeting peptide. Accordingly, a hexahistidine tag for chromatographic purification was positioned on the side opposite to the RGD peptide (Figure 2A). For the expression of recombinant apoptins in *E. coli* cells, we constructed plasmids in which the chimeric genes encoding apoptins with RGD peptides and His-tags were under the control of an inducible T7 phage late gene promoter. These plasmids were used to transform cells of two *E. coli* expression strains: BL21(DE3)Gold and Rosetta2(DE3), followed by optimization of cultivation conditions to achieve the highest accumulation of recombinant proteins. Different temperatures (+28 °C/+37 °C), various inducers (lactose, IPTG), and induction times (overnight/4 h) were analyzed. The presence of the target protein in the cells was analyzed by electrophoresis in a 15% SDS-PAGE. Depending on the cultivation and induction conditions, recombinant apoptins accumulated both in the soluble fraction (in small amounts) and in the inclusion body fraction. Ultimately, the Rosetta2(DE3) strain was selected, and the optimal cultivation protocol was induction with IPTG at +37 °C for four hours. This protocol ensured maximum accumulation of recombinant proteins in inclusion bodies. The yields of the target recombinant proteins ranged from 2 to 5 mg per 1 L of bacterial culture (protein quantification was performed after chromatographic purification). Subsequently, the inclusion body fraction of bacterial cells was obtained, and the target proteins were isolated using metal-chelate chromatography under denaturing conditions. The optimal renaturation protocol for the recombinant proteins was a two-step dialysis into a buffer containing 200 mM L-arginine hydrochloride, 50 mM Tris-HCl, pH 7.6. Following renaturation, the solubility of the recombinant proteins was analyzed (Supplementary Figure S1), along with an assessment of their stability at two temperatures: RT and +37 °C (Supplementary Figures S2 and S3). All RGD-apoptins remained stable in the renaturation buffer for at least one week of incubation at both temperatures, with no visible precipitation or degradation observed. Prior to testing the apoptins on human cell cultures, the proteins were sterilized by filtration and concentrated by ultrafiltration. In total, six recombinant apoptins were obtained: four RGD-apoptins (His-Apo-R13, R13-Apo-His, His-Apo-R15, R15-Apo-His) and two control apoptins without RGD peptides (His-Apo and Apo-His) (Figure 2).

### 2.2. Analysis of Cell Viability

**Figure 3:**
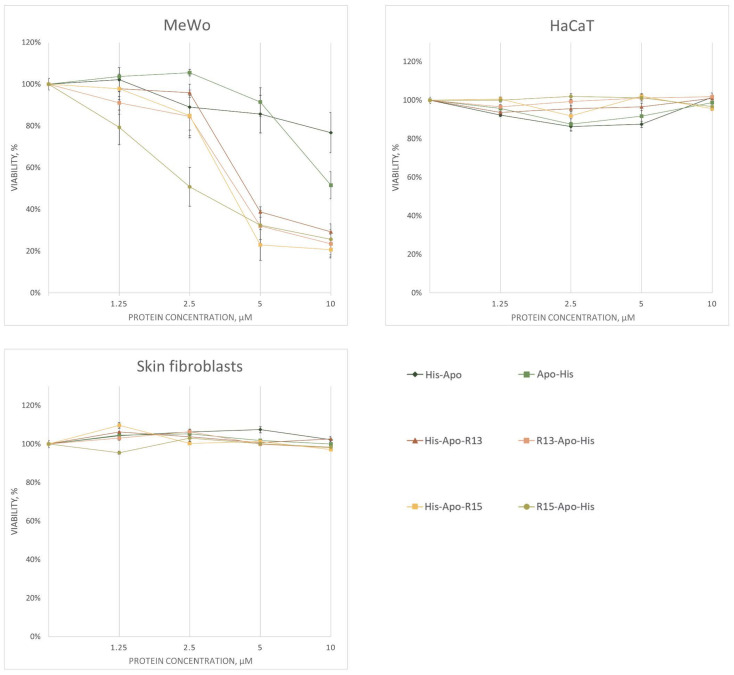
Cell viability analysis of three cell cultures (MeWo, HaCaT, primary skin fibroblasts) 72 h after treatment with RGD-apoptins at four concentrations (1.25 μM, 2.5 μM, 5 μM, and 10 μM). Viability was assessed using the resazurin assay. Data points represent the mean cell viability (as a percentage of the control) ± standard error of three biological replicates. The statistical analysis is provided in the Supplementary Materials (Supplementary Table S1).

The effect of RGD-apoptins on cell viability was tested on three human skin cell cultures: MeWo (malignant melanocytes), HaCaT (transformed keratinocytes), and primary skin fibroblasts. The assessment was performed using the vital dye resazurin. Untreated live cells, as well as cells treated with the renaturation buffer for recombinant proteins containing L-arginine, were used as controls. Cell viability counts after RGD-apoptin treatment were normalized to the renaturation buffer control. Experiments were performed in three biological replicates. All four RGD-apoptins showed a dose-dependent decrease in cell viability of MeWo line after 72 h (Figure 3). The Supplementary Materials section contains a statistical analysis of the differences in cell viability 72 h after the addition of RGD-apoptins (Supplementary Table S1), as well as cell viability data for the 24 h (Supplementary Figure S4) and 48 h (Supplementary Figure S5) time points. The IC50 values were: 4.37 ± 0.11 µM for His-Apo-R13, 3.72 ± 0.63 µM for R13-Apo-His, 3.08 ± 0.35 µM for His-Apo-R15, and 2.53 ± 0.10 µM for R15-Apo-His. Unexpectedly, some reduction in melanoma cell viability was also observed upon addition of the control apoptins without RGD peptides. We discuss the possible reason for this in the Discussion section. In the case of the HaCaT line and primary skin fibroblasts, no decrease in cell viability was observed.

### 2.3. Analysis of Apoptosis Induction

**Figure 4:**
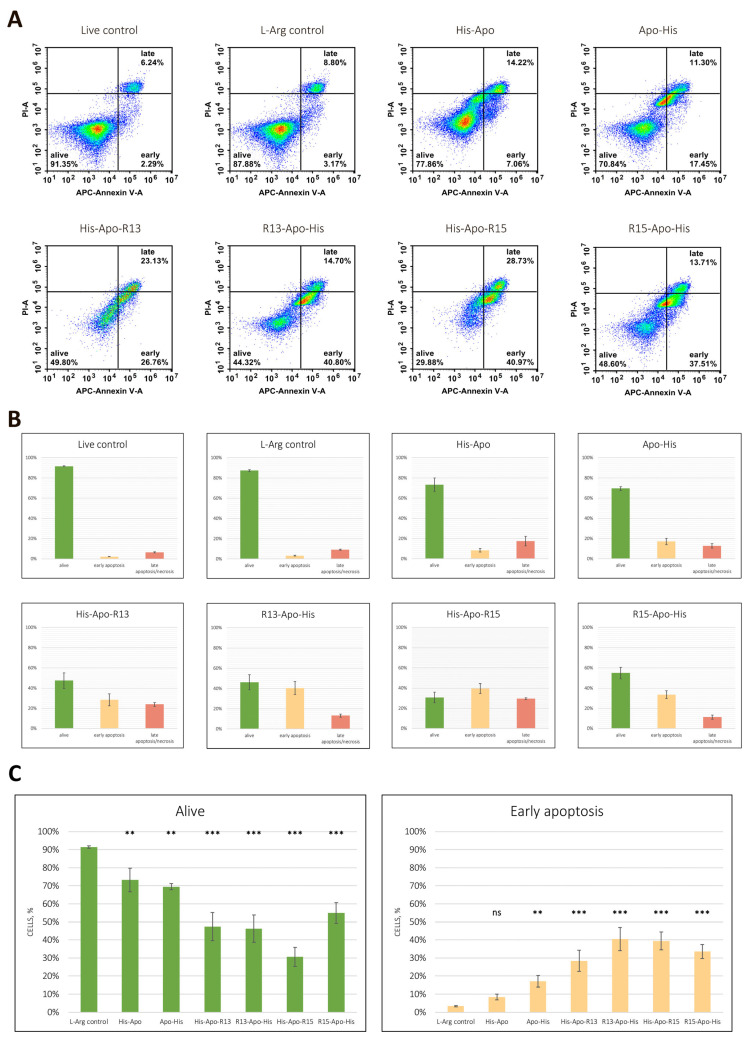
Flow cytometric APC Annexin V/propidium iodide (PI) double staining analysis of apoptosis. (**A**) Representative flow cytometry plots illustrating the percentage of double-negative cells (lower left quadrant), annexin V positive cells (lower right quadrant), and PI/annexin V double-positive cells (upper right quadrant). (**B**) Apoptosis rate determined in three biological replicates. Data are presented as mean ± standard deviation of the percent of live cells (green), cells in early apoptosis (peach), cells in late apoptosis or necrosis (red). (**C**) Statistical analysis of the differences in the number of live cells and cells in early apoptosis relative to the corresponding L-arginine controls. Data are presented as mean ± standard deviation of three biological replicates. Statistical significance markers: **—*p* < 0.01, ***—*p* < 0.001, ns—not significant. Normality was assessed using the Shapiro–Wilk test, and homogeneity of variances was evaluated using Levene’s test. Group differences were analyzed by one-way ANOVA followed by Dunnett’s post hoc test for multiple comparisons versus the control group. Statistical significance was set at *p* < 0.05.

Next, we confirmed that the death of human melanoma cells treated with RGD-apoptins occurs via apoptosis. The induction of programmed cell death was assessed by Annexin V binding to phosphatidylserine, whose externalization to the outer leaflet of the plasma membrane is a characteristic event in the early stages of apoptosis. To differentiate between early and late apoptosis, cells were counterstained with propidium iodide (PI). Cells stained with both Annexin V and PI were considered to be dead either via necrosis or late apoptosis. Cells stained only with Annexin V were considered early apoptotic. Unstained cells were considered viable. The effect of RGD-apoptins was evaluated on the MeWo cell line 24 h after adding the recombinant proteins at a concentration of 10 µM. Two controls were used simultaneously: untreated live cells and live cells treated with the L-arginine renaturation buffer (at the same final concentration as in the recombinant protein solution—24 mM). The experiment was performed in three biological replicates. The number of cells in the early apoptotic stage after treatment with RGD-apoptins was: 28.5 ± 5.9% for His-Apo-R13, 40.4 ± 6.4% for R13-Apo-His, 39.5 ± 5.0% for His-Apo-R15, and 33.6 ± 3.9% for R15-Apo-His (Figure 4). The following results were obtained for the control apoptins: 8.4 ± 1.7% for His-Apo and 17.1 ± 3.3% for Apo-His. In general, the Annexin V test results correlated with the cell viability test: compared to the controls, a slight increase in early apoptotic cells was observed after treatment with the control apoptins, and a significant increase in such cells was observed after treatment with RGD-apoptins.

### 2.4. Transcriptomic Analysis of Human Melanoma Cells After RGD-Apoptin Treatment

**Figure 5:**
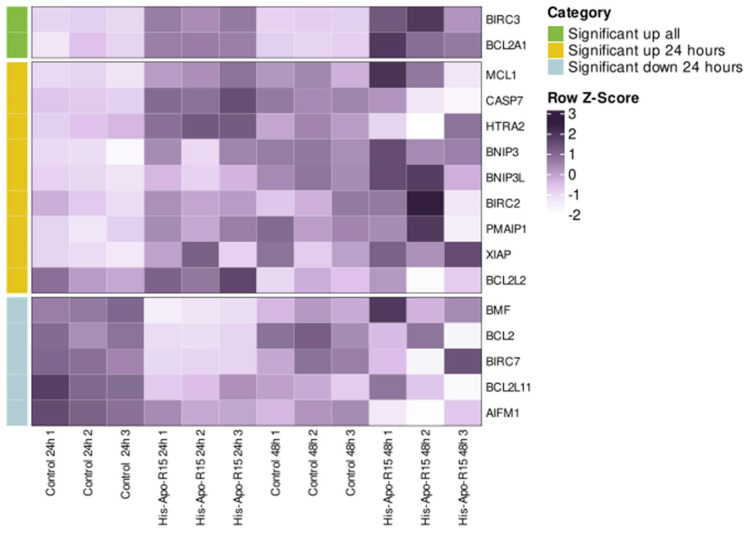
Transcriptomic analysis of MeWo cells treated with His-Apo-R15 (at 24 and 48 h post-treatment). Control cells were treated with the renaturation buffer containing L-arginine. Data are shown for each of the three biological replicates. Heat map showing the expression levels of apoptosis-related genes that were significantly differentially expressed in at least one comparison group (FDR < 0.05). Colors represent row Z-scores based on TPM values, scaled across samples within each gene.

To analyze the effects of RGD-apoptins on gene expression level, we selected the recombinant protein His-Apo-R15, as it showed slightly better results in the Annexin V test compared to the other apoptins. The protein was added to the MeWo cell line at a concentration of 10 µM, and RNA was isolated from the cells after 24 and 48 h. Cells treated with the L-arginine renaturation buffer were used as a control for differential expression analysis. The experiment was performed in three biological replicates. We were primarily interested in genes associated with apoptosis. Accordingly, a set of 45 genes related to both pro- and anti-apoptotic activity was selected for analysis (see Supplementary Table S2 for the full list). For 16 genes from this set, a statistically significant change in expression was observed as early as the first day after the addition of the recombinant protein (Figure 5; see Figure S6 for the heatmap of the full apoptosis-related gene set). Upregulation was observed for five pro-apoptotic genes (*HTRA2, CASP7, PMAIP1, BNIP3, BNIP3L*) and for four anti-apoptotic genes (*BIRC2, XIAP, MCL1, BCL2L2*). Downregulation was detected for three pro-apoptotic genes (*AIFM1, BCL2L11, BMF*) and two anti-apoptotic genes (*BCL2, BIRC7*). Only two apoptosis-related genes, the anti-apoptotic *BIRC3* and *BCL2A1*, showed a significant increase in expression both on day one and day two.

**Figure 6:**
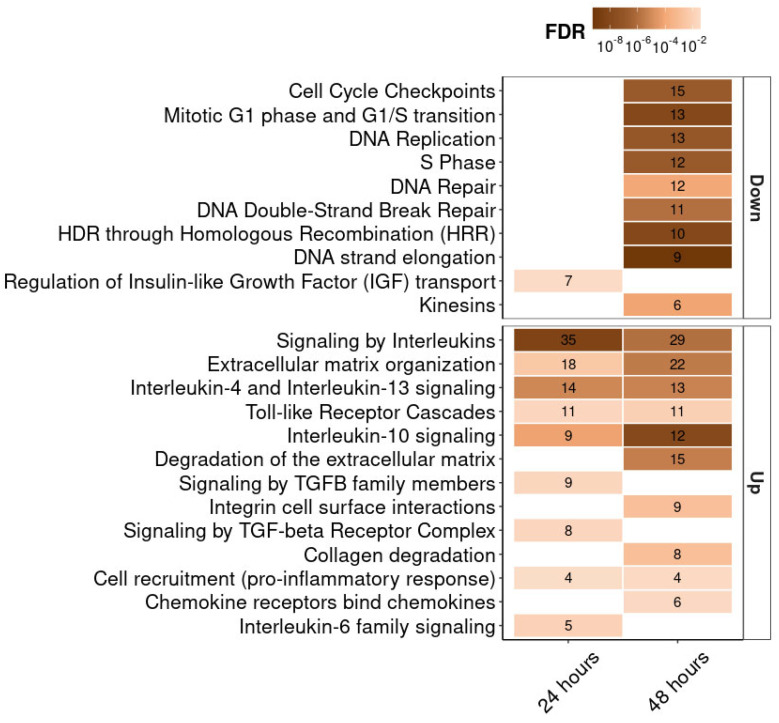
Transcriptomic analysis of MeWo cells treated with His-Apo-R15 (at 24 and 48 h post-treatment). Heat map showing the Reactome pathway enrichment analysis. It shows genes differentially expressed between cells treated with His-Apo-R15 and control cells. The x-axis represents the incubation time after His-Apo-R15 treatment, and the y-axis shows the signaling pathways, ranked by the number of associated genes. The numbers within the boxes indicate the number of genes associated with each pathway at FDR < 0.05.

We also analyzed the differential expression of all genes in our experiment using the Reactome database. The most important signaling pathways, for which the gene expression change compared to the control was statistically significant, are shown in Figure 6. First of all, we note the upregulation of genes associated with extracellular matrix organization and integrin receptors. For 12 such genes, increased expression was observed both at 24 and 48 h (*DCN, LTBP1, NID2, ICAM1, SERPINE1, SDC4, COL8A1, ADAMTS1, MMP14, CTSS, MMP1, COL13A1*). Expression of integrin receptor genes *ITGB8*, *ITGA2*, and *ITGB3* was increased only at 24 h. At 48 h, upregulation was observed for the genes *FN1, LUM, LAMB3, COL7A1, COL9A3, COL16A1*, encoding the extracellular matrix proteins fibronectin, lumican, the beta subunit of laminin, and the alpha chains of collagens type 7, 9, and 16, respectively. Among other signaling pathways, we noted upregulation of interleukin genes (*IL1B, IL6, IL11, IL24, IL32*), interleukin receptors genes (*IL1R1, IL4R, OSMR*), and associated genes (*MEF2A, SOCS3, SEBPD*) at both 24 and 48 h. Expression of some genes involved in inflammation and Toll-like receptor signaling pathways was also elevated (*NFKBIA, NFKB2, MEF2A, MAP3K8, IRAK2, LY96, CTSS, FOS, JUN, PELI1, C3, NT5E*). Significant downregulation of the genes associated with DNA replication, transcription, and repair, and with cell cycle regulation (*MCM10, MCM2, ORC1, MCM5, GINS1, RFC5, GINS2, RPA1, PCNA, DNA2, SKP2, PRIM1, H4C12, RAD51, RAD51AP1, RAD51D, XRCC3, EME1, EXO1, TYMS, CDCA8, CDC25A, RRM2, GTSE1, ZWINT, CENPO, ERCC6L, DTL*) was observed at 48 h. *MITF* gene expression analysis showed the following results: 105 ± 7 TPM and 98 ± 4 TPM for control cells at 24 and 48 h, respectively, and 75 ± 5 TPM and 73 ± 25 TPM for cells treated with RGD-apoptins at 24 and 48 h, respectively. On a logarithmic scale, the values fell in the range between log2 TPM = 6.2 and log2 TPM = 6.7. Changes in *MITF* expression in cells treated with recombinant protein were not statistically significant compared to control cells.

## 3. Discussion

The primary goal of our work was to develop recombinant proteins with dual specificity for tumor cells. There are few examples of such molecules in the scientific papers, primarily due to the limited number of proteins that induce tumor cell death without harming normal cells. These include the viral proteins apoptin, E4orf4 (derived from adenovirus), and NS1 (derived from parvovirus), as well as nonviral proteins TRAIL, mda7 (IL24), and HAMLET [16,25]. All of the above-mentioned polypeptides already possess specific oncolytic activity, but by additionally linking them to a peptide or protein targeting a tumor-specific receptor, their selectivity for malignant cells can be increased. Interestingly, apoptin has been most often used in the development of dual-specific anticancer drugs. Thus, Niesler and colleagues created a fusion protein consisting of apoptin linked to epidermal growth factor [26]. A somewhat different approach to creating therapeutic drugs of this type was demonstrated by Li et al., who created a recombinant adenovirus containing the apoptin gene under the control of a tumor-specific promoter [27]. We, in turn, decided to generate chimeric proteins consisting of apoptin and RGD peptides, which have long been used in the design of anticancer drugs. To produce RGD-apoptins, we used a heterologous *E. coli* expression system, which is simple to use and allows for the production of significant quantities of target proteins within a short timeframe.

Testing of the obtained recombinant RGD-apoptins on the MeWo cell line revealed a dose-dependent decrease in the viability of malignant melanocytes with similar IC50 values for all four proteins. In theory, the two control apoptins should not have exhibited a cytotoxic effect on these cells due to the absence of RGD peptides in their sequence. Nevertheless, some decrease in the viability of MeWo cells was observed in this case as well. After careful analysis of the native apoptin amino acid sequence, we discovered an RGD-like tripeptide, RTD (arginine–threonine–aspartic acid) at positions 77–79. There are some contradictory reports regarding the binding of peptides with this amino acid sequence to αVβ6- [28,29,30] and αVβ3-integrins [31]. Although it is unclear whether the RTD-containing region of apoptin can adopt the correct conformation for binding to integrins, the presence of this tripeptide in the protein may explain the effects we observed upon exposure of MeWo cells to control apoptins. HaCaT keratinocytes and primary skin fibroblasts showed no reduction in viability when exposed to RGD-apoptins. HaCaT cells are transformed and therefore should be sensitive to apoptin, as was previously demonstrated in [14]. However, their membrane lacks beta-3 integrin molecules, meaning RGD-apoptins should not bind to these cells and should not exert an apoptogenic effect on them.

Annexin V assay confirmed that the reduced viability of malignant melanocytes exposed to RGD-apoptins was due to apoptosis. Control apoptins also induced apoptosis, but to a significantly lesser extent. Analyzing the results of the annexin V and resazurin assays, it can be concluded that the location of the RGD peptide relative to the apoptin molecule (at the N- or C-terminus) does not affect the apoptogenic effect of the chimeric proteins. Differences in the amino acid sequences of the RGD peptides also did not significantly affect the cytolytic activity of the proteins. It should be noted, however, that we demonstrated the pro-apoptotic effect of RGD-apoptins in malignant melanocytes with the wild-type *BRAF* gene. Nevertheless, a significant proportion of melanoma cases (44.9% according to Greaves et al. [32]) harbor *BRAF* mutations, the most frequent of which is the T1799A transversion, resulting in a valine-to-glutamic acid substitution (V600E) in the protein. This mutation leads to constitutive activation of the MAPK (mitogen-activated protein kinase) pathway, which promotes enhanced cell proliferation. Melanomas with *BRAF* mutations are generally considered more aggressive and more therapy-resistant [33]. Therefore, it is crucial to further investigate the pro-apoptotic activity of RGD-apoptins in this type of malignant melanocyte as well.

Transcriptomic analysis helps to elucidate the apoptotic processes induced by RGD-apoptins in malignant melanocytes. Using two time points after the addition of recombinant His-Apo-R15 (24 and 48 h), we were able to dynamically evaluate gene expression in tumor cells following protein exposure. Remarkably, most changes in the expression of apoptosis-related genes occurred in the first 24 h. Most notably, we detected upregulation of the pro-apoptotic genes *CASP7* and *HTRA2*. The *CASP7* gene encodes caspase-7, one of three effector caspases activated at the final stage of the caspase cascade during programmed cell death. HTRA2 (also known as Omi) is a key protein in the mitochondrial apoptotic pathway [34]. This serine protease cleaves a number of inhibitors of apoptosis from the IAP family, specifically XIAP, cIAP1 (the *BIRC2* gene product), and cIAP2 (the *BIRC3* gene product). XIAP cleavage, in turn, leads to the activation of caspases-3, -7, and -9. Interestingly, in our experiment, the genes encoding the above-mentioned target proteins of the HTRA2 protease, namely *XIAP*, *BIRC2*, and *BIRC3*, showed increased expression. This may indicate a compensatory response of tumor cells to the reduced levels of proteins encoded by these genes as a result of the proteolytic action of HTRA2. Based on this fact, it can be hypothesized that the anti-apoptotic proteins XIAP, cIAP1, and cIAP2 are important for melanoma survival. Three more pro-apoptotic genes, (*PMAIP1, BNIP3*, and *BNIP3L*) upregulated in response to the addition of RGD-apoptin, encode minor components of the apoptotic machinery. PMAIP1 (also known as Noxa) belongs to the BH3-only subfamily of the Bcl2 family proteins and is known to inhibit the action of two prosurvival proteins, MCL1 and Bfl1/A1 (the *BCL2A1* gene product) [35]. Again, as with the HTRA2 targets, we observed the upregulation of the *MCL1* and *BCL2A1* genes, which encode proteins targeted by PMAIP1. Two more BH3-only proteins, BNIP3 and BNIP3L (also known as Nix), are localized on the outer membrane of mitochondria and may be involved in the processes of apoptosis induction, as well as in mitophagy (a specialized type of autophagy that leads to the destruction of damaged mitochondria) [36].

Among the downregulated apoptosis-related genes, *BCL2*, which encodes the key regulator of the intrinsic pathway of programmed cell death, should be highlighted first. Although it remains unclear how exactly RGD-apoptin contributes to the downregulation of this gene, there is no doubt that a decrease in Bcl-2 protein levels should significantly shift the balance toward apoptosis. Another anti-apoptotic gene whose expression was downregulated in our experiment is *BIRC7*. This gene encodes the apoptosis inhibitor BIRC7 (also known as livin or ML-IAP) from the IAP family, and, like all members of this family, contains a BIR motif in its structure. BIRC7 inhibits caspases -3, -7 and -9, thereby blocking apoptosis in the cell. Notably, elevated levels of this protein are observed in most melanoma cell lines, which may indicate the importance of BIRC7 for the survival of this tumor type [37].

We would like to specifically focus on the expression analysis of the *BCL2A1* and *BIRC3* genes. We previously mentioned that their upregulation may result from a compensatory response by tumor cells to the action of their antagonists. However, the fact that increased expression of these two genes is still detectable 48 h after the addition of RGD-apoptin (unlike the other apoptosis-associated genes analyzed) reflects, in our view, their crucial role in the response of melanoma cells to therapeutic intervention. Previously, Haq and colleagues demonstrated that *BCL2A1* is a melanoma-specific oncogene and likely plays a key role in the development of resistance to BRAF inhibitors [38]. More recently, Glasheen and colleagues showed that melanoma cells with and without BRAF mutations have elevated levels of the cIAP2 protein (the *BIRC3* gene product), which mediates tumor cell resistance to MEK inhibitors [39]. These data confirm that BCL2A1 and cIAP2 proteins can be considered key players in the development of drug resistance in melanoma cells. Nevertheless, despite the upregulation of *BCL2A1* and *BIRC3* observed in our experiment, the majority of melanoma cells died, suggesting that RGD-apoptins may be able to bypass the defense mechanisms of these tumor cells.

Transcriptomic analysis of all genes in melanoma cells mostly confirmed the range of effects we expected from recombinant RGD-apoptin. A significant portion of upregulated genes were associated with integrin receptors and the extracellular matrix. Thus, after 24 h, the production of integrin subunits β3, β8, and α2 increased. In our opinion, this may indicate a compensatory response of the tumor cell to the internalization (and/or blockade) of αVβ3 integrins bound to recombinant His-Apo-R15. The integrin receptor family is characterized by functional redundancy, whereby blockade of one receptor is compensated by another from the same family. Therefore, it is quite logical that in addition to increased expression of the beta-3 subunit, the tumor cell additionally synthesizes subunits of other integrins (β8 and α2). This is also evidenced by the increased expression of the ligands of these integrins—collagen, laminin, and fibronectin. Downregulation of the genes associated with DNA replication, transcription, and repair, as well as cell cycle regulation, 48 h after the addition of RGD-apoptin, clearly indicates the onset of the execution phase of apoptosis in melanoma cells. This may be a consequence of either direct binding of apoptin to DNA or the final stage of the intrinsic mitochondrial pathway that triggers cell death.

We also analyzed the expression of the *MITF* gene, which encodes a melanocyte-specific transcription factor playing a key role in the proliferation, differentiation, and survival of these cells [40]. *MITF* expression levels are often used for the phenotypic characterization of melanomas. Elevated expression is observed in proliferating or differentiating melanocytes, while low expression is characteristic of cells with increased invasive potential. Furthermore, *MITF* can promote melanocyte survival by upregulating the expression of the anti-apoptotic genes *BCL2*, *BCL2A1*, and *BIRC7*. In our experiment, no statistically significant differences in *MITF* expression were observed in cells treated with RGD-apoptins compared to the control. The overall expression level of this transcription factor in our model system can be defined as intermediate, characteristic of melanomas with a proliferative phenotype (*MITF* expression levels were classified according to Singh et al. [41]). In summary, it can be stated that the addition of RGD-apoptins to melanoma cells does not significantly affect *MITF* expression, yet it successfully overcomes the pro-survival effect regulated by this transcription factor.

Taken together, our transcriptomic data show that melanoma cells treatment with recombinant RGD-apoptin leads to activation of apoptosis primarily via a Bcl-2-dependent mechanism. However, drug resistance mechanisms are simultaneously enhanced in tumor cells, primarily due to increased production of the apoptosis regulators BCL2A1 and cIAP2. The goal of anticancer therapy is to shift the balance between apoptosis-promoting signals and tumor cell survival efforts. In our experiments, this balance shifts toward the death of malignant melanocytes, suggesting that RGD-apoptins may be promising molecules for drug development.

## 4. Materials and Methods

### 4.1. Bacterial Strains, Human Cell Cultures, Plasmids, Virus

*E. coli* strains Top10, BL21(DE3)Gold, and Rosetta2(DE3); pET-based plasmids (Novagen, Madison, WI, USA) with modified multiple cloning sites (MCS) and pTZ57R plasmid (Fermentas, Vilnius, Lithuania) were used in the study. To evaluate the functional activity of the recombinant proteins, human melanoma cell line MeWo (ATCC HTB-65^TM^, Manassas, VA, USA), human keratinocyte cell line HaCaT and primary skin fibroblast culture obtained from a 26-year-old female donor, were used. The HaCaT cell line and primary skin fibroblast culture were kindly provided by Dr. M.A. Lagarkova (Federal Research and Clinical Center of Physical-Chemical Medicine, Moscow, Russia). The use of donor material was approved by the Ethics Committee of the Federal Research and Clinical Center of Physical-Chemical Medicine on 1 June 2021 (protocol No. 2021–06-01/1). Donor material was obtained in accordance with the principles of the Declaration of Helsinki. All cell cultures were maintained in DMEM medium (Servicebio, Wuhan, China) supplemented with 10% FBS (HyClone Cytiva, Logan, UT, USA) in humidified atmosphere with 5% CO_2_ at +37 °C; for primary skin fibroblasts, recombinant FGF-2 (lab made) at a final concentration of 4 ng/mL was added to the medium. Chicken anemia virus (CAV) strain was kindly provided by the All-Russian Research Veterinary Institute of Poultry Science (St. Petersburg, Russia).

### 4.2. Construction of Recombinant Plasmids

Viral DNA was isolated using a standard phenol/chloroform extraction method. The nucleotide sequence encoding the apoptin ORF was amplified by PCR using primers Apo-For and Apo-Rev (Table 1) and CAV DNA as a template, and was cloned into the pTZ57R vector. Chimeric genes encoding apoptins fused with RGD peptides were obtained using a two-round PCR. To generate genes encoding apoptin with a C-terminal RGD peptide, primer pairs Apo-For and Apo-R13-1r (or Apo-R15-1r) with the pTZ57R/apoptin plasmid as a template were used in the first round of PCR. Primer pairs Apo-For and Apo-R13-2r (or Apo-R15-2r) with the DNA fragment from the first round as a template were used in the second round of PCR. The resulting chimeric gene was cloned into the pET15mcs vector (which contains a sequence encoding a hexahistidine tag upstream of the MCS) using BamHI and SalI restriction sites. To generate genes encoding apoptin with an N-terminal RGD peptide, primer pairs R13-Apo-1f (or R15-Apo-1f) and Apo-Rev with the pTZ57R/apoptin plasmid as a template were used in the first round of PCR. Primer pairs R13-Apo-2f (or R15-Apo-2f) and Apo-Rev with the DNA fragment from the first round as a template were used in the second round of PCR. The resulting chimeric gene was cloned into the pETmin vector (which contains a sequence encoding a hexahistidine tag downstream of the MCS) using BamHI and SalI restriction sites. Two constructs encoding apoptin without additional peptides, with a His-tag at either the N- or C-terminus, were also assembled.

### 4.3. Production of Recombinant Proteins in E. coli

*E. coli* Rosetta2(DE3) cells were transformed with plasmids encoding the recombinant chimeric apoptins, plated on Petri dishes with LB agar containing ampicillin (150 µg/mL) and chloramphenicol (30 µg/mL), and incubated overnight at +37 °C. A single colony from the transformation plate was inoculated into a 100 mL LB medium containing antibiotics at the appropriate concentrations and incubated in a shaker–incubator at +37 °C and 225 rpm overnight. The resulting culture (25 mL aliquots) was transferred into four 1 L flasks, each containing 250 mL of LB with ampicillin and chloramphenicol, and grown at +37 °C with shaking at 225 rpm until an optical density (OD_600_) of 0.6 was reached. Upon reaching the required density, IPTG was added to a final concentration of 1 mM, and cultivation continued for another 4 h. After incubation, cells were centrifuged for 15 min at 6000× *g* at +4 °C, the pellet was washed with cold Phosphate-Buffered Saline (PBS), and centrifuged again under the same conditions. The bacterial cell pellet was then resuspended in a buffer containing 100 mM NaCl, 10 mM Tris-HCl, 1 mM EDTA, 0.1% Triton X-100, 0.1 mg/mL lysozyme, pH 7.0, subjected to ultrasonication, and centrifuged at maximum speed (11,000 rpm/15,557× *g*) at +4 °C for 10 min. Inclusion bodies were obtained by washing the pellet three times with 1% Triton X-100 (Sigma, St. Louis, MO, USA), followed by centrifugation at maximum speed. The resulting pellet was dissolved in a chromatographic buffer containing 8 M urea (neoFroxx, Einhausen, Deutschland).

### 4.4. Chromatographic Purification and Renaturation of Recombinant Proteins

The inclusion body solution was treated with a Qsonica Q700 ultrasonic disintegrator (Qsonica L.L.C, Newtown, CT, USA) for 60 s at 35% amplitude and centrifuged at 50,000× *g* for 30 min. The supernatant was loaded onto a Tricorn 10/50 column with Ni Sepharose Fast Flow resin (GE Healthcare, Chicago, IL, USA) equilibrated with Solution AM (8 M urea, 20 mM Tris-HCl, 500 mM NaCl, 10 mM imidazole, pH 7.5). After loading, the column was washed with 20 mL of Solution AM, and the bound proteins were eluted with Solution EM (8 M urea, 20 mM Tris-HCl, 500 mM NaCl, 500 mM imidazole, pH 7.5). The flow-through was collected and reloaded onto another Tricorn 10/50 column with Ni Sepharose High Performance resin (GE Healthcare, Chicago, IL, USA), equilibrated with Solution AM. This column was washed with 20 mL of Solution AM, and the bound proteins were eluted with Solution EM. Chromatography was performed using an NGC system (Bio-Rad, Hercules, CA, USA). The flow rate for both columns was 1 mL/min. Process monitoring and fraction collection were based on online measurement of eluate absorbance at 280 nm. The presence of the target protein in the collected fractions was determined by SDS-PAGE with Coomassie G-250 (Serva Electrophoresis GmbH, Heidelberg, Germany) gel staining.

Renaturation of the isolated recombinant proteins was achieved by a two-step dialysis using SnakeSkin 3.5K dialysis tubing (Thermo Scientific, Waltham, MA, USA). The proteins were dialyzed from Solution EM into a buffer containing 500 mM L-arginine hydrochloride, 50 mM Tris-HCl, pH 7.6, and then into the final buffer containing 200 mM L-arginine hydrochloride, 50 mM Tris-HCl, pH 7.6. The resulting protein solution was sterilized by filtration through 0.22 µm CA filters (Yicozoo, Xi’an, China) and concentrated by ultrafiltration using Amicon Ultra-15 3 kDa centrifugal filters (Merck Millipore, Burlington, MA, USA).

### 4.5. Cell Viability Test

Cell viability was assessed using a resazurin assay according to the manufacturer’s instructions. Cells were seeded in 96-well plates and maintained in DMEM (Servicebio, Wuhan, China) with 10% FBS (HyClone Cytiva, Logan, UT, USA) until they reached 70% confluency. The medium was then removed, and 100 μL of Opti-MEM medium (Gibco, Carlsbad, CA, USA) containing recombinant apoptins at four concentrations: 1.25, 2.5, 5, and 10 μM were added to the wells. After 24, 48, and 72 h, resazurin (Chem-Impex International, Wood Dale, IL, USA) was added to a final concentration of 250 μM and the cells were incubated in a CO_2_ incubator for 2 h. Fluorescence was detected on a Tecan Infinite 200pro plate reader (Tecan Austria GmbH, Grödig, Austria) at an excitation wavelength of 560 nm and an emission wavelength of 590 nm. Each experiment was performed in triplicate.

### 4.6. Apoptosis Assay

The assay was performed according to the work of Romashin et al. [42] with some modifications. Briefly, the cells were seeded into wells of 24-well plate and maintained in DMEM with 10% FBS until they reached 70% confluency. The medium was then replaced with OptiMEM containing recombinant apoptins at a concentration of 10 μM, and cells were incubated for 24 h. Cells were harvested by trypsinization, rinsed twice with HBSS (Capricorn Scientific, Ebsdorfergrund, Germany) and incubated with a mixture of propidium iodide (Servicebio, Wuhan, China) and APC Annexin V (Thermo Fisher Scientific, Waltham, MA, USA) for 15 min in dark. The cells were analyzed immediately after incubation via a NovoCyte Flow Cytometer (Agilent Technologies, Santa Clara, CA, USA). NovoExpress (v1.6.2) software was used for data analysis (Agilent Technologies, Santa Clara, CA, USA).

### 4.7. Preparation of Transcriptomic Libraries and RNA Sequencing

RNA was extracted using ExtractRNA reagent (Evrogen, Moscow, Russia), according to the manufacturer protocol. Remaining genomic DNA was removed by Turbo DNAse (Invitrogen, Carlsbad, CA, USA). RNA quality was assessed on a Tape Station microfluidic analyzer using the High Sensitivity RNA kit (Agilent Technologies, Santa Clara, CA, USA). The RNA Integrity Number (RIN) was greater than 7 in all samples. Total RNA (400 ng) was used for library preparation. Enrichment of polyadenylated RNA was performed with KAPA mRNA Capture Kit (Roche, Basel, Switzerland) and libraries were prepared using the KAPA RNA Hyper Kit (Roche, Basel, Switzerland), according to the manufacturer protocol. Subsequently, RNA cleanup was performed with the RNA Clean XP kit (Beckman Coulter, Brea, CA, USA). The library underwent a final cleanup using the KAPA HyperPure Beads (Roche, Basel, Switzerland) after which the libraries size distribution and quality were assessed using a high sensitivity DNA chip (Agilent Technologies, Santa Clara, CA, USA). Libraries were pooled equimolarly and diluted to a final concentration of 750 pM. Sequencing was carried out on a NextSeq 1000 platform (Illumina, San Diego, CA, USA) using the NextSeq 1000/2000 P2 Reagents kit (200 Cycles) v3, with 4% PhiX (Illumina, San Diego, CA, USA) added as an internal control. The raw data have been deposited in the SRA database: https://www.ncbi.nlm.nih.gov/bioproject/PRJNA1346047 (accessed on 17 October 2025).

### 4.8. Transcriptome Data Analysis

Quality filtering of the sequence reads was performed using Trimgalore (v. 0.6.6) and Trimmomatic (v. 0.39) software [43]. Transcript-level abundances were quantified for each sample using a quasi-mapping approach with Salmon (v. 1.5.1) [44] on the Gencode reference transcriptome GRCh38.p13 [45] with default parameters. Then transcript-level abundances were aggregated to the gene-level abundances using the Tximport R package (v. 1.34.0) [46]. Differential expression analysis was performed with DESeq2 (v 1.42.1) [47] using the Wald test. To correct for multiple comparisons, the Benjamini–Hochberg method (FDR) was used. Changes in expression were considered significant if FDR < 0.05, |Log2FC| ≥ 0.7, and the gene reached a cumulative expression of >6 TPM in at least three samples of the relevant group. Functional Reactome pathways enrichment analysis was performed using the clusterProfiler R package (v. 4.6.2) [48] and the ReactomePA R package (v. 1.42.0) [49]. FDR cutoff of 0.05 were considered as statistically significant.

## 5. Conclusions

In this study, we described the generation and evaluation of activity of RGD-apoptins, novel recombinant proteins with potential for human melanoma therapy. These proteins induced apoptosis in cultured malignant melanocytes at micromolar concentrations. Exposure of keratinocytes and skin fibroblasts to similar concentrations of RGD-apoptins did not reduce cell viability. Transcriptomic analysis of malignant melanocytes 24 and 48 h after treatment with recombinant RGD-apoptin allowed us to highlight signaling pathways associated with apoptosis induction and the tumor cell response to therapeutic intervention. This is the first report on these chimeric proteins, and we briefly outline future research directions. First and foremost, it will be of interest to determine how efficiently RGD-apoptins are internalized by cells and how they traffic intracellularly. Second, the mechanism by which apoptins lacking the RGD peptide enter melanoma cells remains unclear. Is it mediated by the RTD motif in their amino acid sequence and the possible use of integrins for internalization, or do apoptins bind to an alternative cell-surface receptor? Third, the pro-apoptotic activity of RGD-apoptins needs to be tested in *BRAF*-mutated melanoma cells and in normal melanocytes. And last but not least, to comprehensively evaluate the therapeutic potential of RGD-apoptins in vivo, their efficacy must be validated in animal models, specifically, in mice bearing human melanoma xenografts.

## Figures and Tables

**Table 1:** Primers used for PCR.

Primer Name	Sequence (5′-3′)
Apo-For	acggatccaacgctctccaagaagatactc ^1^
Apo-Rev	atagtcgaccagtcttatacaccttcttgcg
Apo-R13-1r	accacggcagccgctcagtgcacccgccagtcttatacaccttcttgc
Apo-R13-2r	atagtcgactcactgctggaagcaatcaccacggcagccgctcag
Apo-R15-1r	ggcaacctgggaagccatctgcgcctgccagtcttatacaccttcttgc
Apo-R15-2r	atagtcgactcattcctgactgcaatcaccacggcaacctgggaagccat
R13-Apo-1f	gtggtgattgcttccagcagaacgctctccaagaagatactc
R13-Apo-2f	acggatccctgagcggctgccgtggtgattgcttccagcag
R15-Apo-1f	ccgtggtgattgcagtcaggaaaacgctctccaagaagatactc
R15-Apo-2f	acggatccgatggcttcccaggttgccgtggtgattgcagtcag

^1^ The restriction sites used in plasmid assembly are underlined.

## Data Availability

The data presented in this study are openly available in SRA, https://www.ncbi.nlm.nih.gov/bioproject/PRJNA1346047 (accessed on 17 October 2025), [PRJNA1346047].

## Supplementary Materials

The following supporting information can be downloaded at: https://www.mdpi.com/article/10.3390/ijms262412016/s1.

## Abbreviations

The following abbreviations are used in this manuscript:

RGD	arginine–glycine–aspartic acid
RTD	arginine–threonine–aspartic acid
CAV	chicken anemia virus
MCS	multiple cloning site
ORF	open reading frame
IPTG	isopropyl beta-D-thiogalactoside
SRA	Sequence Read Archive
FDR	false discovery rate
TPM	transcripts per million
PI	propidium iodide
IAP	Inhibitors of apoptosis proteins
BH3	Bcl-2 homology 3
BIR	Baculovirus Inhibitor of apoptosis protein Repeat
