# Supplementary material for: Recombinant RGD-Apoptins Decrease Human Melanoma Cell Viability
Source: Int J Mol Sci. 2025 Dec 13;26(24):12016. doi: 10.3390/ijms262412016 (PMC12732338; doi:10.3390/ijms262412016)
Supplement: Supplementary file 1 [file ijms-26-12016-s001.zip › ijms-3992411-supplementary.pdf]

Supplementary materials for article
"Recombinant RGD-apoptins decrease human melanoma cell viability"

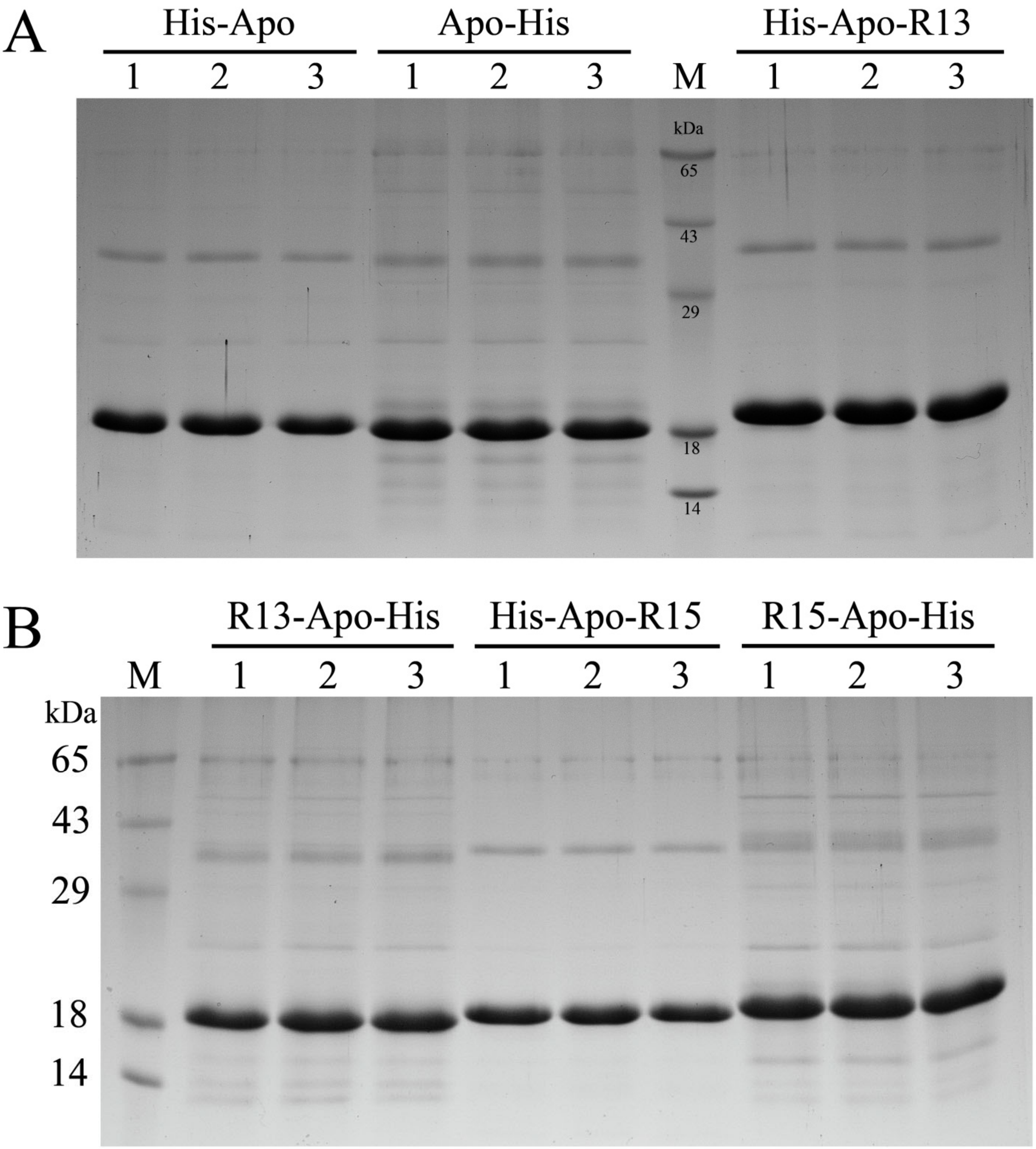

Figure S1. Solubility analysis of recombinant RGD-apoptins after two-step dialysis. For all RGD-apoptins (panels A and B): lane 1 – protein in elution buffer (8 M urea, 20 mM Tris-HCl, 500 mM NaCl, 500 mM imidazole, pH 7.5); lane 2 – protein in renaturation buffer (200 mM L-arginine HCl, 50 mM Tris-HCl, pH 7.6), suspension after vigorous mixing; lane 3 – protein in renaturation buffer (200 mM L-arginine HCl, 50 mM Tris-HCl, pH 7.6), supernatant after centrifugation at 14100g for 10 minutes. All protein samples were applied in equivalent volumes (5 microliters).

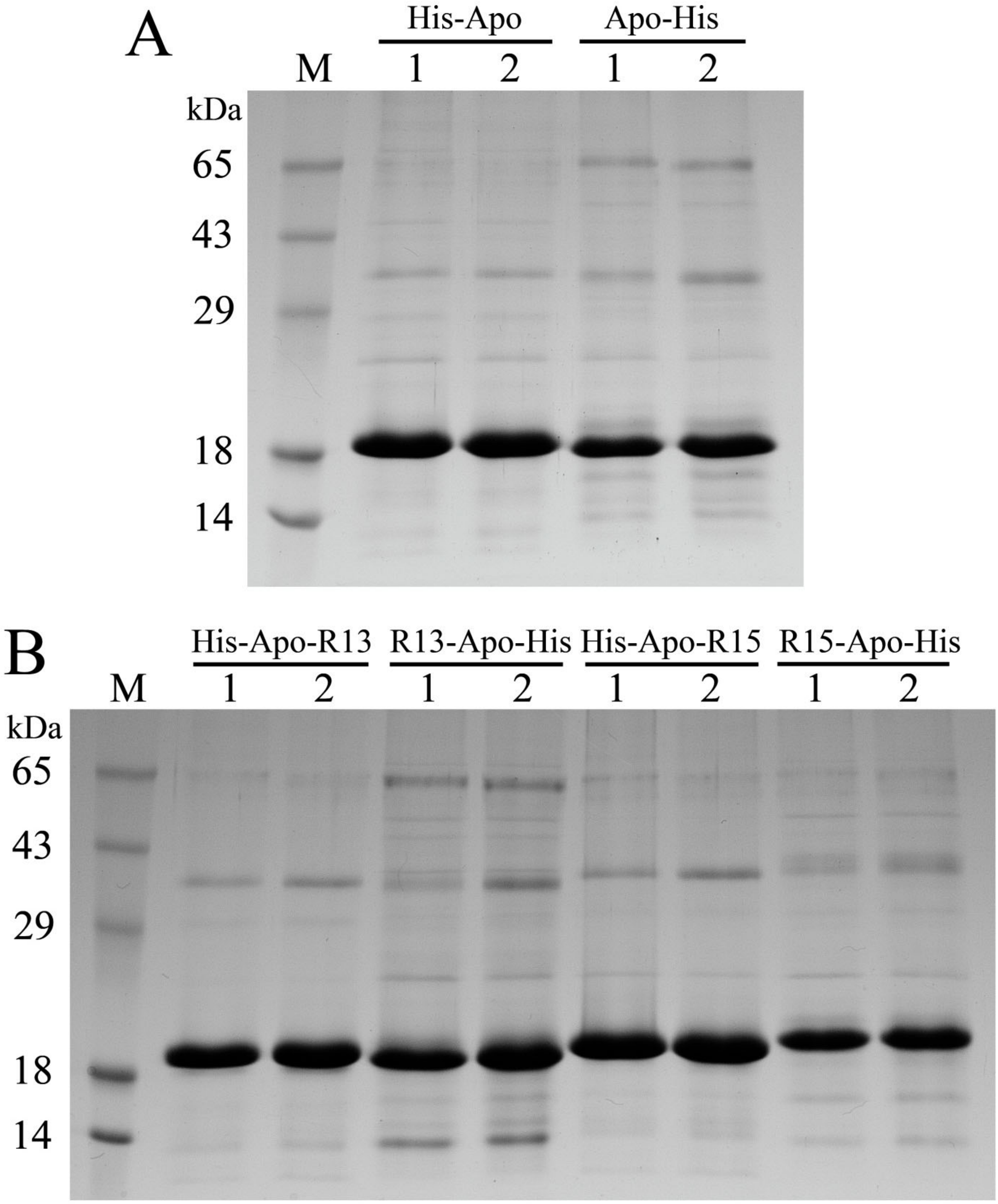

Figure S2. Stability analysis of recombinant RGD-apoptins at room temperature over one week. For all RGD-apoptins (panels A and B): lane 1 – protein in renaturation buffer (200 mM L-arginine HCl, 50 mM Tris-HCl, pH 7.6) before incubation; lane 2 – protein in renaturation buffer (200 mM L-arginine HCl, 50 mM Tris-HCl, pH 7.6) after incubation over a 7-day period at room temperature, supernatant after centrifugation at 14100g for 10 minutes. All protein samples were applied in equivalent volumes (5 microliters).

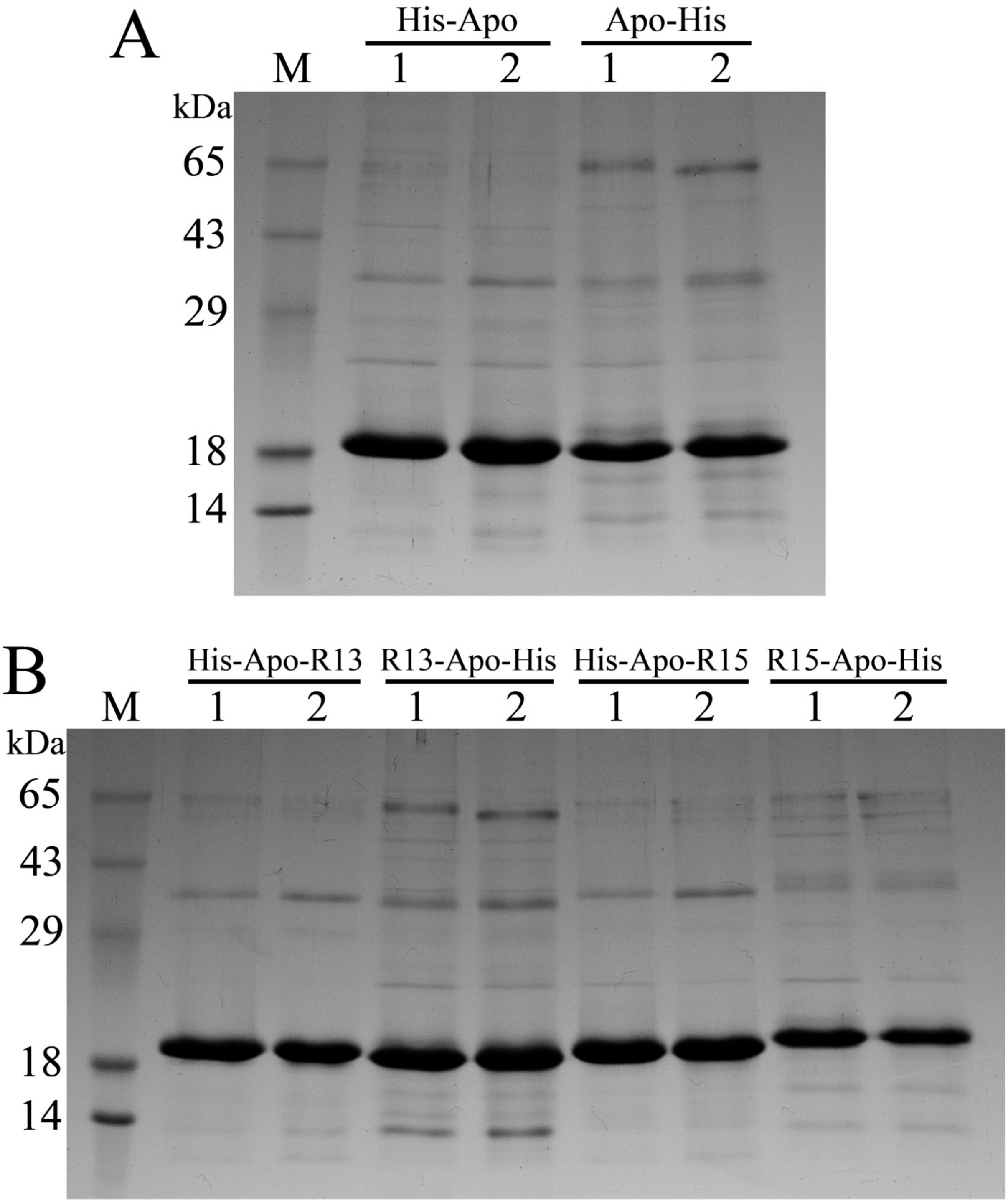

Figure S3. Stability analysis of recombinant RGD-apoptins at +37°C over one week. For all RGD-apoptins (panels A and B): lane 1 – protein in renaturation buffer (200 mM L-arginine HCl, 50 mM Tris-HCl, pH 7.6) before incubation; lane 2 – protein in renaturation buffer (200 mM L-arginine HCl, 50 mM Tris-HCl, pH 7.6) after incubation over a 7-day period at +37°C, supernatant after centrifugation at 14100g for 10 minutes. All protein samples were applied in equivalent volumes (5 microliters).

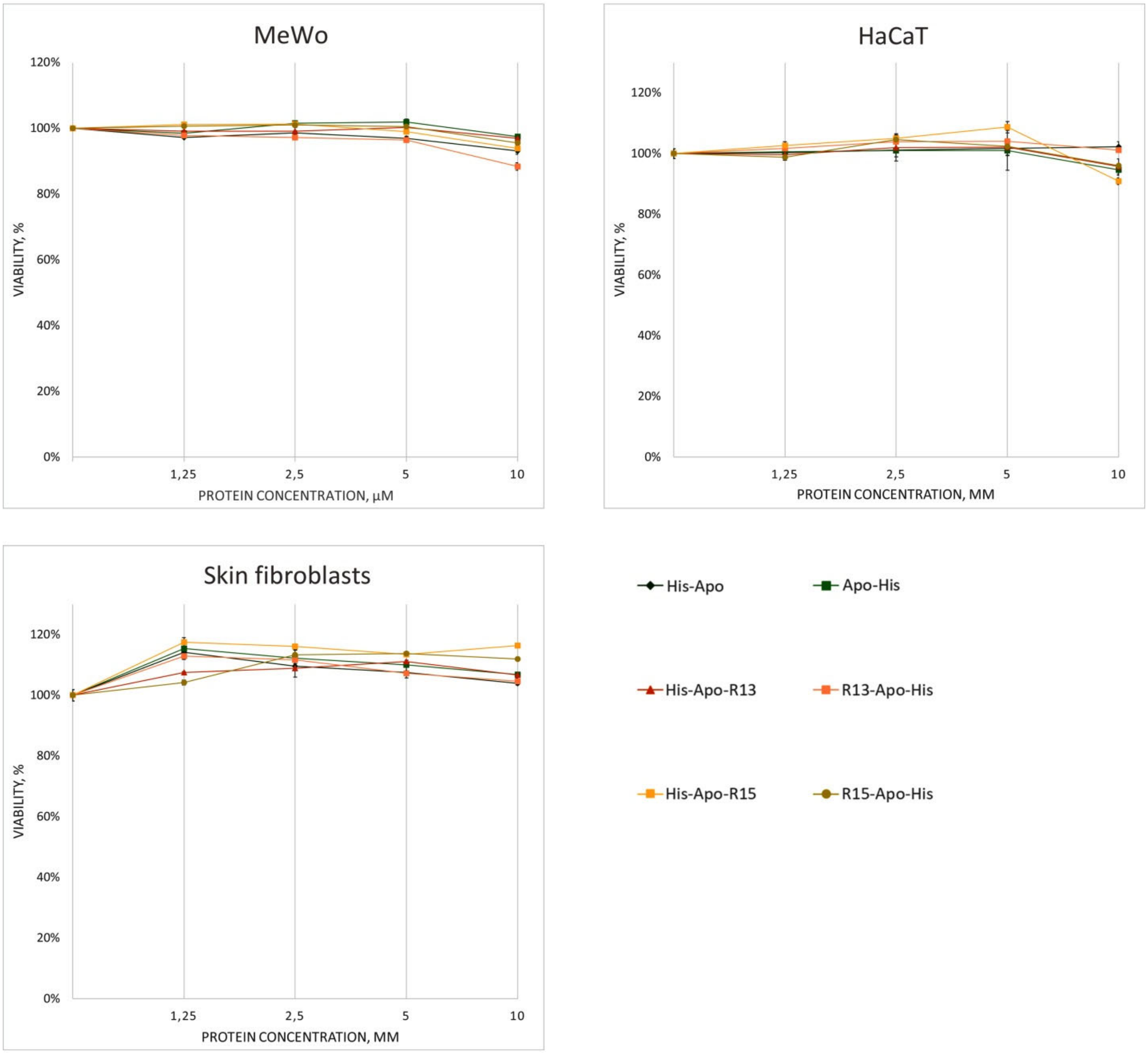

Figure S4. Cell viability analysis of three cell cultures (MeWo, HaCaT, primary skin fibroblasts) 24 hours after treatment with RGD-apoptins at four concentrations (1.25 µM, 2.5 µM, 5 µM, and 10 µM). Viability was assessed using the resazurin assay. Data points represent the mean cell viability (as a percentage of the control) ± standard error of three biological replicates.

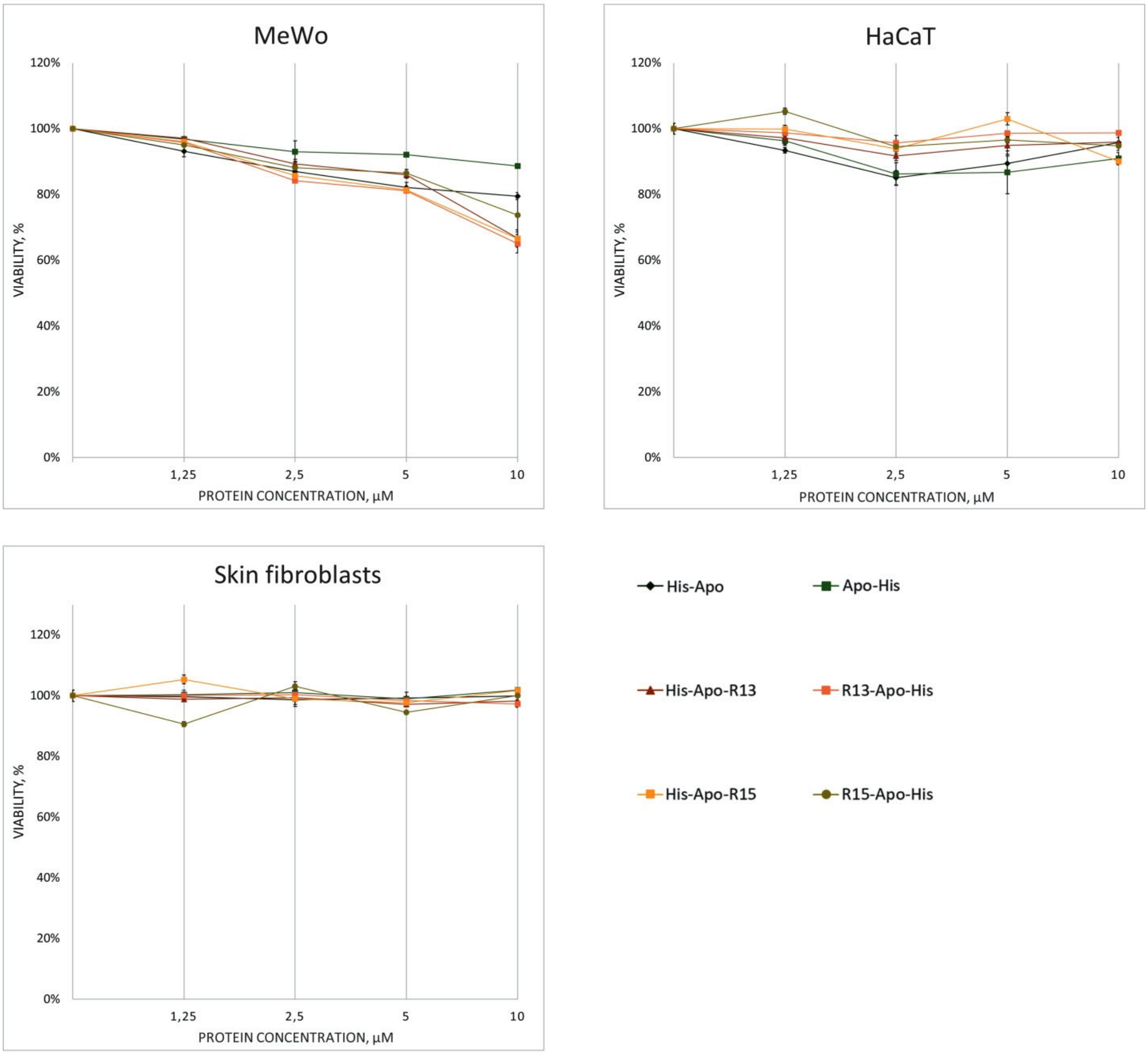

Figure S5. Cell viability analysis of three cell cultures (MeWo, HaCaT, primary skin fibroblasts) 48 hours after treatment with RGD-apoptins at four concentrations (1.25 µM, 2.5 µM, 5 µM, and 10 µM). Viability was assessed using the resazurin assay. Data points represent the mean cell viability (as a percentage of the control) ± standard error of three biological replicates.

	protein	1.25 µM	2.5 µM	5 µM	10 µM
**MeWo**	His-Apo	0.9953 (ns)	0.8532 (ns)	0.6273 (ns)	0.3739 (ns)
	Apo-His	0.7437 (ns)	0.1953 (ns)	0.6179 (ns)	0.0351 (*)
	His-Apo-R13	0.9883 (ns)	0.9998 (ns)	0.0004 (***)	0.0018 (**)
	R13-Apo-His	0.6481 (ns)	0.7322 (ns)	0.0404 (*)	0.0120 (*)
	His-Apo-R15	0.9943 (ns)	0.8145 (ns)	0.0151 (*)	0.0004 (***)
	R15-Apo-His	0.3537 (ns)	0.0943 (ns)	0.0173 (*)	0.0175 (*)
**HaCaT**	**protein**	**1.25 µM**	**2.5 µM**	**5 µM**	**10 µM**
	His-Apo	0.0945 (ns)	0.0404 (*)	0.0262 (*)	0.9161 (ns)
	Apo-His	0.4107 (ns)	0.1796 (ns)	0.5698 (ns)	0.9805 (ns)
	His-Apo-R13	0.2469 (ns)	0.3823 (ns)	0.6971 (ns)	0.9970 (ns)
	R13-Apo-His	0.5316 (ns)	0.9989 (ns)	0.0949 (ns)	0.8163 (ns)
	His-Apo-R15	0.9981 (ns)	0.0965 (ns)	0.8304 (ns)	0.3348 (ns)
	R15-Apo-His	0.3945 (ns)	0.8829 (ns)	0.9732 (ns)	0.6465 (ns)
**Skin fibroblasts**	**protein**	**1.25 µM**	**2.5 µM**	**5 µM**	**10 µM**
	His-Apo	0.4020 (ns)	0.2193 (ns)	0.1542 (ns)	0.7858 (ns)
	Apo-His	0.4280 (ns)	0.2867 (ns)	0.8583 (ns)	1.0000 (ns)
	His-Apo-R13	0.2192 (ns)	0.8055 (ns)	0.9932 (ns)	0.7455 (ns)
	R13-Apo-His	0.6514 (ns)	0.2033 (ns)	1.0000 (ns)	0.8775 (ns)
	His-Apo-R15	0.0706 (ns)	0.9995 (ns)	0.9466 (ns)	0.6291 (ns)
	R15-Apo-His	0.3364 (ns)	0.7093 (ns)	1.0000 (ns)	0.9152 (ns)

Table S1. Statistical analysis of the differences in cell viability in three human cell cultures (MeWo, HaCaT, primary skin fibroblasts) 72 hours after the addition of recombinant apoptins at four concentrations (1.25 μM, 2.5 μM, 5 μM, 10 μM) compared to control. Data are presented as p-values with statistical significance markers: * — $p < 0.05$, ** — $p < 0.01$, *** — $p < 0.001$, ns – not significant. Normality of distributions and homogeneity of variances were assessed using the Shapiro–Wilk and Levene's tests, respectively. Because variance homogeneity was not satisfied for every dataset, group comparisons were performed using Welch ANOVA followed by the Games–Howell post-hoc test, which does not assume equal variances. Statistical significance was set at $p < 0.05$.

Pro-apoptotic genes	Anti-apoptotic genes
APAF1, CYCS, DIABLO, HTRA2, AIFM1, ENDOG<br>Caspase family: *CASP1, CASP2, CASP3, CASP4, CASP5, CASP6, CASP7, CASP8, CASP9, CASP10*<br>Bcl-2 family: *BAX, BAK1, BAD, BIK, HRK, BID, BOK, BMF, BBC3, PMAIP1, BNIP3, BNIP3L, BCL2L11, BCL2L14*<br>*BCL2L1**, *BCL2L13**	IAP family: *NAIP, XIAP, BIRC2, BIRC3, BIRC5, BIRC6, BIRC7*<br>Bcl-2 family: *BCL2, BCL2L2, BCL2A1, BCL2L10, BCL2L12, MCL1*<br>*BCL2L1**, *BCL2L13**

Table S2. Complete list of apoptosis-related genes used in the transcriptome data analysis. The genes are divided into pro- and anti-apoptotic. Products of genes marked with * can exhibit both activities: inducing and inhibiting apoptosis.

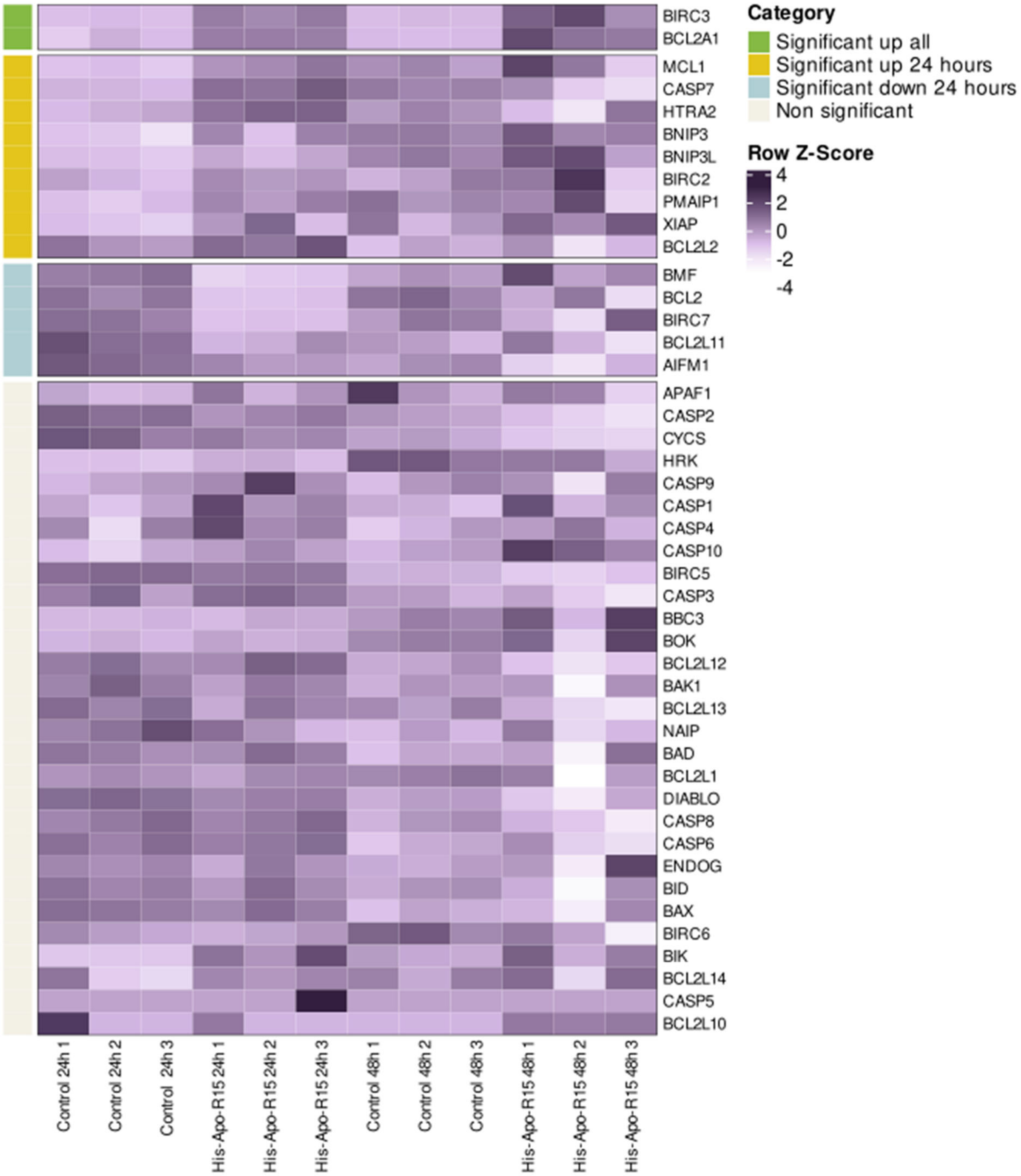

Figure S6. Transcriptomic analysis of MeWo cells treated with His-Apo-R15 (at 24 and 48 hours post-treatment). Control cells were treated with the renaturation buffer containing L-arginine. Data are shown for each of the three biological replicates. Heat map showing the expression levels of apoptosis-related genes. Colors represent row Z-scores based on TPM values, scaled across samples within each gene.
